# A randomised controlled trial investigating the causal role of the medial prefrontal cortex in mediating self-agency during speech monitoring and reality monitoring

**DOI:** 10.21203/rs.3.rs-3280599/v1

**Published:** 2023-09-11

**Authors:** Songyuan Tan, Yingxin Jia, Namasvi Jariwala, Zoey Zhang, Kurtis Brent, John Houde, Srikantan Nagarajan, Karuna Subramaniam

**Affiliations:** University of California San Francisco Medical Center; University of California San Francisco Medical Center; Palo Alto University; University of California San Francisco Medical Center; University of California San Francisco Medical Center; University of California San Francisco Medical Center; University of California San Francisco Medical Center; University of California San Francisco Medical Center

**Keywords:** self-agency, speech monitoring, reality monitoring, medial prefrontal cortex, repetitive transcranial magnetic stimulation

## Abstract

Self-agency is being aware of oneself as the agent of one’s thoughts and actions. Self agency is necessary for successful interactions with the external world (reality-monitoring). The medial prefrontal cortex (mPFC) is considered to represent one neural correlate underlying self-agency. We investigated whether mPFC activity can causally modulate self-agency on two different tasks involving speech-monitoring and reality-monitoring. The experience of self-agency is thought to result from being able to reliably predict the sensory outcomes of one’s own actions. This self-prediction ability is necessary for successfully encoding and recalling one’s own thoughts to enable accurate self-agency judgments during reality-monitoring tasks. This self-prediction ability is also necessary during speech-monitoring tasks where speakers compare what we hear ourselves say in auditory feedback with what we predict we will hear while speaking. In this randomised-controlled study, heathy controls (HC) are assigned to either high-frequency transcranial magnetic stimulation (TMS) to enhance mPFC excitability or TMS targeting a control site. After TMS to mPFC, HC improved self-predictions during speech-monitoring tasks that predicted improved self-agency judgments during different reality-monitoring tasks. These first-in-kind findings demonstrate the mechanisms of how mPFC plays a causal role in self-agency that results from the fundamental ability of improving self-predictions across two different tasks.

## Introduction

Self-agency is of cardinal importance because it underlies self-awareness in the context of interacting with the external world (i.e., reality monitoring) (Subramaniam, 2021). The experience of self-agency is thought to result from being able to reliably predict the sensory outcomes of one’s own actions (Subramaniam, 2021; Subramaniam et al., 2018). Compared to external actions, the expected sensory outcomes of one’s own actions are highly predictable (Chang et al., 2013; Korzyukov et al., 2017; Niziolek et al., 2013). This self-prediction ability is necessary for successfully encoding and recalling one’s own thoughts to enable accurate self-agency judgments (i.e., accurate recognition of self-generated information) during reality-monitoring tasks (Subramaniam et al., 2019). This self-prediction ability is also necessary during speech-monitoring tasks where speakers consistently compare what we hear ourselves say in our auditory feedback with what we predict we will hear while speaking (Subramaniam et al., 2018).

Prior research studies have revealed that healthy controls (HC) show increased activity in the medial prefrontal cortex (mPFC) when they make lower-level self-predictions while performing speech-monitoring tasks (Ranasinghe et al., 2019; Subramaniam, 2021), as well as making higher-level self-agency judgments during reality-monitoring tasks (Subramaniam et al., 2012; Subramaniam et al., 2019). In our prior reality-monitoring tasks, in which participants had to differentiate self-generated from externally-derived information, we found increased mPFC activity while HC encoded and retrieved self-generated information, which correlated with their ability to make accurate self-agency judgments, revealing that the mPFC represents a neural substrate underlying self-agency Subramaniam et al., 2012; Subramaniam et al., 2019). In our speech-monitoring tasks, the sense of self-agency results only when auditory feedback slightly diverges from expectations of what speakers expect to hear when they speak (Subramaniam et al., 2018; Subramaniam, 2021). When HC hear these small deviations in auditory feedback when speaking, they judge the pitch perturbations as errors in their speech output and make corrective responses to compensate for these speech errors (Burnett et al., 1998; Chang et al., 2013; Liu and Larson, 2007; Subramaniam et al., 2019). These corrective responses are modified by the amount of confidence/reliance that subjects place on self-predictions about their speech output - the greater the reliance on their self-predictions, the smaller the reliance on the external perturbed auditory feedback. This induces subjects to make smaller corrective responses, and results in their enhanced sense of self-agency that arises from subjects placing increased reliance on their own self-predictions to control their own speech outcome rather than relying on external auditory feedback. In our prior speech monitoring studies, HC exhibited increased activity in mPFC when they made smaller corrective responses upon hearing perturbations to their pitch in auditory feedback, revealing that they showed increased reliance on following their self-predictions about their expected speech output (Ranasinghe et al., 2019; Subramaniam, 2021).

Across convergent evidence from functional imaging studies (magnetoencephalography (MEG), functional magnetic resonance imaging (fMRI), electroencephalography (EEG)) and single neuron studies, subjects show increased activity in the mPFC prior to self-generated actions, but not before externally-perceived actions (Cabeza and St Jacques, 2007; Fried et al., 2011; Keller and Heckhausen, 1990; Khalighinejad et al., 2018; Passingham et al., 2010; Subramaniam et al., 2012; Subramaniam et al., 2019). These convergent results suggest that mPFC likely represents one neural site that mediates the self-prediction computations that results in the experience of self-agency. Given these correlative data that mPFC supports both self-predictions and self-agency in HC, we now test whether mPFC activity can causally modulate this self-prediction ability to impact self-agency on two different tasks of reality and speech monitoring. This will show that mPFC provides a unitary basis for self-agency, driven by reliance on self-predictions. Here, we use repetitive transcranial magnetic stimulation (rTMS) as a causal neurostimulation tool to test whether increasing mPFC excitability with high-frequency 10Hz rTMS, will enhance self-predictions during speech monitoring to predict better self-agency judgments during reality monitoring. Specifically, in the present study, we implemented a double-blinded randomised controlled trial (RCT) in which HC are assigned to either active rTMS to enhance mPFC excitability or 10Hz rTMS applied to a control site outside the self-agency network (N = 15). Using reality and speech monitoring tasks, measured from pre-to-post rTMS, we examined the causal mechanisms of how active rTMS modulation of mPFC activity induces changes in the self-agency network in HC, compared to baseline and control rTMS.

We had three specific hypotheses: (1) Compared to control rTMS, enhancing mPFC excitability by active 10Hz rTMS would induce HC to make reduced corrective responses when HC heard small pitch perturbations in their auditory feedback. Put another way, we hypothesized that after rTMS to mPFC, HC would make reduced corrections when they heard these small pitch perturbations in their auditory feedback, which would indicate their greater reliance on their self-predictions to control their speech outcome and their enhanced sense of self-agency. (2) Compared to control rTMS, enhancing mPFC excitability by active 10Hz rTMS would improve improved judgments of self-agency (i.e., correct identification of self-generated information) while HC perform the reality monitoring task. (3) Smaller corrective responses induced by rTMS applied to mPFC would predict improved judgments of self-agency while HC perform the reality monitoring task. If these hypotheses are confirmed, the present study would provide the first causal evidence for mPFC activity underlying a collective sense of self-agency that is driven by the basic mechanism of HC making reliable self-predictions during a speech monitoring task, which potentiates self-agency judgments while they perform a reality monitoring task.

## Methods

### Participants and procedures

In the present double-blinded randomised controlled trial (RCT), 30 healthy control participants (20 males, 10 females, mean age = 43 years, mean education = 17 years) volunteered to participate in this study at the University of California San Francisco (UCSF). All methods and procedures were performed in accordance with the guidelines of the Internal Review Board (IRB) at UCSF and were approved by the IRB at UCSF. This study was first registered on 19/03/2021 at clinicaltrial.gov (NCT04807530). HC participants were recruited from March 2021 to October 2022 through our clinicaltrial.gov site (NCT04807530) or from our previous research studies if they had consented to be contacted for future studies. Due to the COVID-19 pandemic, we first completed recruitment and all data acquisition for HC subjects in order to ensure safety and tolerability of all study procedures, before moving on to recruitment of more vulnerable schizophrenia patient populations. All subjects were told that the purpose of the study was to examine how TMS affects the sense of self that is involved during speech, mood and thinking (cognition). After providing written consent to volunteer to participate in the study, participants were evaluated by a clinical psychologist and completed questionnaires to meet the inclusion criteria. Inclusion criteria for healthy control participants (HC) were absence of neurologic psychiatric disorders (Axis I or Axis II (SCID-Nonpatient edition)) and major illnesses, no current or history of substance dependence or abuse, meets MRI criteria, good general physical health, age between 18 and 64 years, right-handed, and English as the first language. All participants provided written informed consent for this study and then completed structural magnetic resonance imaging (MRI), a speech-monitoring task and a reality-monitoring task at baseline. After getting informed consent, each subject had a deidentified number code that was provided to the study team by the research assistant (RA) who administered the rTMS. HC were matched at a group level on age, gender, and education, and then in this parallel RCT design, half the subjects were randomly assigned to either the active 10Hz rTMS condition targeting mPFC (n=15), or the control 10Hz rTMS condition targeting the temporoparietal site outside the self-agency network (n=15) (Table 1). Only the RA responsible for randomization who also administered rTMS (the rTMS RA) was aware of the condition to which each subject is assigned and did not discuss randomization with anyone, ensuring the double-blind RCT. This rTMS RA used a block randomization method to randomize subjects into groups that resulted in equal sample sizes. Individual subject data was analyzed by the outcomes assessor (who only joined this lab after all the data were acquired) using each deidentified subject code number supplied by the rTMS RA. For the group analyses, the rTMS RA organized subjects into the active versus control condition with color labels (e.g. condition purple and condition green) in order for the outcomes assessor to complete all group analyses for each condition. Only once all the data analyses were fully completed for all HC, the colour condition blind was removed to reveal which condition was the active and control condition, in order to examine the effects of the active rTMS and control rTMS on speech-monitoring and reality-monitoring tasks. One participant who was assigned to the active rTMS condition was not available to complete rTMS. Informed consent, and all speech-monitoring and reality-monitoring data and TMS acquisition were acquired at the Biomagnetic Imaging Laboratory (BIL) on the Parnassus campus at UCSF: https://radiology.ucsf.edu/research/labs/biomagnetic-imaging. Primary outcomes included: magnitude of corrective responses induced by pitch perturbations during speech-monitoring, self-agency judgments (% accuracy for self-generated identification), and overall reality-monitoring performance (d-prime scores).

### Speech monitoring task

Participants performed a speech monitoring task at baseline and immediately after the rTMS session. Participants wore a microphone (AKG Pro Audio C520 Professional Head-Worn Condenser Microphone, AKG Acoustics, Vienna, Austria) and a pair of headphones. The microphone was attached to an amplifier that was connected to a Dell computer sound card (M-Audio Delta 44 4×4 analog I/O, M-Audio, Cumberland, RI). The amplified audio signal was played back via the headphones. Participants confirmed they could clearly hear the audio signal prior to the experiment.

The speech-monitoring pitch perturbation experiment was programmed in Matlab, and consisted of 9 runs constituting 15 trials per run, totaling 135 trials. Each trial began with a green dot that appeared on the computer screen. Participants were instructed to vocalize the vowel / / when they saw the green dot. They continued phonation for 2.5 seconds until the dot disappeared while listening to real-time auditory feedback from the headphones. In each trial, the phonation onset triggered a brief perturbation (of 100 cents or 1/12^th^ of an octave) in the pitch of each participant’s auditory feedback (Kort et al., 2014; Ranasinghe et al., 2017; Subramaniam, 2021; Subramaniam et al., 2018) for a duration of 400ms following a variable delay between 200–500ms from phonation onset. The direction of the pitch shift was randomised so that it was either upward or downward so that half the trials had a positive shift and the other half had a negative shift. This jittered perturbation and pseudo-random distribution minimized expectation/anticipatory bias, preventing participants from being able to predict either the onset or direction of the pitch shift. The inter-trial interval was 2.5s during which time participants viewed a blank screen.

**Figure 1:**
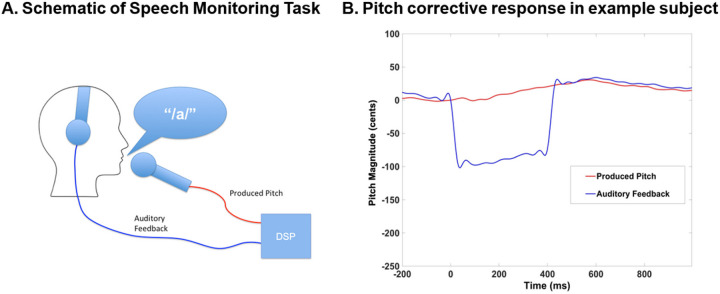
**A**. During the speech monitoring task, participants produced a steady-state /a/ vowel. Their speech was picked up by a microphone and passed through a digital signal processing (DSP) program that provided auditory feedback of their speech in real-time that they heard in the headphones. In each trial of the experiment, the DSP was directed to alter the pitch of the participant’s speech feedback by +/− 100 cents. **B**. Example of one trial from one participant, in which the participant raised their pitch to partly compensate for the effects of the DSP which perturbed the participant’s vocal feedback by lowering the pitch for 400ms by 100 cents.

Pitch perturbation was achieved by utilizing a real-time speech feedback alteration technique implemented by a digital signal processing (DSP) program (see also Raharjo et al., 2021) (Figures 1A–B). The DSP program received the participant’s vocalization as input, which was captured by an optical microphone (Phone-Or Ltd., Or-Yehuda, Israel). The output generated by the DSP program was then delivered back to the participant through earphones (model EAR-3A, Etymotic Research, Inc., Elk Grove Village, IL). The DSP program employed a vocoder process that decomposed incoming speech into pitch and spectral envelope characteristics, and either raised or lowered the pitch of each participant’s outgoing speech in real-time by 100 cents.

### Speech monitoring pitch perturbation analyses

For each participant and for each trial, the raw audio data were examined to extract the pitch time course using an autocorrelation-based pitch tracking method (Parsons, T.W. 1987. Voice and Speech Processing. Mcgraw-Hill College, Blacklick, OH). Participants who had noisy data with pitch tracking errors or incomplete utterances within the analysis interval were excluded (n=3 from the active rTMS condition, and n=2 from the control rTMS condition). An analysis interval of 1200ms was extracted for each trial, which spanned from −200ms to 1000ms relative to the perturbation onset, and was converted from hertz to cents, indicating the deviation from the pre-perturbation baseline. For each participant, the pitch track of each trial was processed by calculating the deviation from the mean pitch track, which was averaged across all trials, encompassing both upward and downward pitch perturbations. The responses to both upward and downward perturbations were combined into a unified dataset representing the absolute magnitude of the response for each participant. Participants responded to applied pitch perturbations by making corrective responses and deviating from their baseline pitch track. For each trial we computed the magnitude of pitch deviations in each participant, by calculating the peak corrective response with respect to the baseline pitch value prior to perturbation. For each participant, we then extracted the mean peak response across trials, as we have performed in our prior studies (Chang et al., 2013; Ranasinghe et al., 2017; Subramaniam, 2021; Subramaniam et al., 2018). Compensatory corrective responses were defined as a mean pitch peak response which opposed the direction of the applied pitch perturbation shift, and yielded positive values. In other words, if the pitch shift was upward, and the response was downward, this denotes a positive compensatory response. Similarly, if the pitch shift was downward and the response was upward, this also denotes a positive compensatory response. The peak magnitude of the corrective responses was pooled together across the upward pitch perturbation shift trials and downward pitch perturbation shift trials. Consistent with our earlier studies (Demopoulos et al., 2018; Ranasinghe et al., 2017; Subramaniam et al., 2018), these analyses techniques examined response deviations from the mean response track and thus the magnitudes of vocal responses to both the upward and downward pitch feedback perturbations were the same distance from baseline and did not change as a function of stimulus direction.

### Reality monitoring task

**Figure 2:**
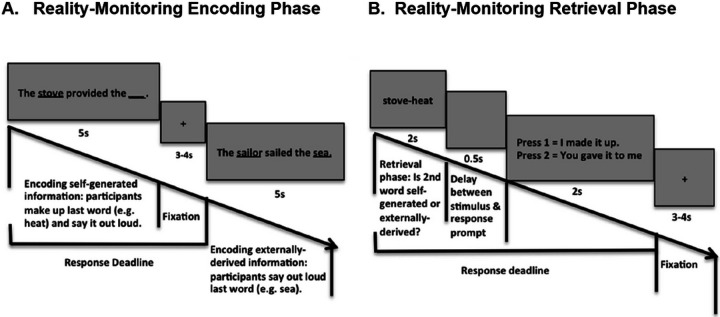
Reality Monitoring Task Design. **A**. During the encoding phase, for half the sentences, the final word was either left blank for participants to make up themselves (e.g., The stove provided the __) or was externally-given by the experimenter (e.g., The sailor sailed the sea). **B**. During the retrieval phase, participants were randomly presented with the noun pairs from the sentences (e.g., stove-heat), and had to identify with a button-press whether the second word was previously self-generated or externally-derived.

Participants in the study performed a reality monitoring task at baseline and immediately after the rTMS session. As described in previous studies (Subramaniam, 2021; Subramaniam et al., 2019; Subramaniam et al., 2020; Subramaniam et al., 2018; Subramaniam et al., 2012), the reality monitoring task consisted of an encoding phase and a memory retrieval phase (see also Figures 2A–B). All participants completed eight runs, with 20 trials per run, totaling 160 trials for the whole task. During the encoding phase, participants were visually presented with semantically-constrained sentences with “noun-verb-noun” structures. For half the sentences, the final word was either left blank for participants to make up themselves (e.g., The *stove* *provided the*__), or was externally-given by the experimenter (e.g., The *sailor* *sailed the* *sea*) (Figure 2A). For each sentence, participants were told to pay attention to the underlined nouns for a subsequent memory test and to vocalize only the final word of each sentence. After the encoding phase, participants then completed the memory retrieval phase where they were randomly presented with the underlined noun pairs from the sentences (e.g., *stove-heat*), and were asked to identify whether the second word was previously self-generated or externally-derived using a button box. At each time-point (i.e., baseline and after rTMS), the sentences were completely different, containing different sets of matched semantically-constrained sentences.

### Reality monitoring analyses

For each participant, self-agency accuracy was computed as the percentage of correctly-identified self-generated items out of the total number of self-generated trials on the reality monitoring task. Signal detection theoretic d-prime analyses were performed to compute overall reality monitoring accuracy. This was done by calculating the hit rate and the false alarm rate for self-generated and externally-presented items, then converting each measure to z-scores, and subtracting the false alarm rate from the hit rate in order to differentiate sensitivity during accurate performance from response bias.

### rTMS Protocol

Our TMS system is a state-of-the-art Nexstim Neuronavigated Brain Stimulator (Helsinki, Finland). This system integrates the TMS figure-8 shaped coil with a navigational system with several landmarks on each subject’s scalp surface matched to their anatomical locations on the 3D MRI that allows for highly accurate cortical targeting individualized to each subject’s MRI anatomy. Once the targeted stimulation site is selected, the system calculates the strength of the electric field in real-time that is incident on the targeted_cortical site (Petrov et al., 2017; Ruohonen and Ilmoniemi, 1999). The specific site that we target in subjects assigned to the active rTMS condition, is based on our prior functional localization of medial frontal activity mediating self-agency (i.e., accurate identification of self-generated information) during reality-monitoring tasks across convergent fMRI and MEG studies (Subramaniam et al., 2012; Subramaniam et al., 2019). We have previously shown that this site can be directly modulated with rTMS to induce significant improvement in self-agency judgments during reality-monitoring (Subramaniam et al., 2020). For subjects assigned to the control rTMS condition, we target the posterior temporoparietal site, which mediates general attention/salience to sensory information but considered to be an independent site outside of the direct self-agency network (Behrmann et al., 2004). In our previous study (Subramaniam et al., 2020), we had established the optimal rTMS dosage parameters that maximized tolerability/comfort in the present study. For both control and active rTMS conditions, we use the same rTMS protocol parameters. The present rTMS parameters have also been deemed safe by the rTMS Consensus Guidelines (Rossi et al., 2009). The rTMS session consisted of 120 trains of 20 pulses (2s duration of 10Hz) for 20 mins at 110% Resting Motor Threshold based on hand electromyography (Subramaniam et al., 2020), which we and others have previously shown to maximize safety and efficacy, without a single_adverse event (Bentwich et al., 2011; Lee et al., 2016; Rabey et al., 2013). Due to prior reports of more discomfort and pain with higher-frequencies from our prior pilot study (Subramaniam et al., 2020), we did not use frequencies of more than 10 Hz or theta-burst stimulation, or longer protocols (single train durations >2s).

### Statistical analyses

The sample size in each group was selected based on our prior reality-monitoring, speech monitoring and randomised controlled TMS studies (Subramaniam et al., 2012; 2018; 2019; 2020). Repeated-measures ANOVAs were implemented to examine group differences, after rTMS compared to baseline, in peak deviation during the 100 cents pitch perturbations, as well as in self-agency judgments (i.e., self-generated identification accuracy) and overall reality monitoring performance (d-prime score). Pearson’s two-tailed correlation tests were used to measure the strength of the linear relationship between peak deviation for 100 cents pitch shifts with self-agency judgments and reality-monitoring performance. Effect sizes (Cohen’s d) were used to quantify the power of the group differences and linear relationships.

## Results

**Figure 3:**
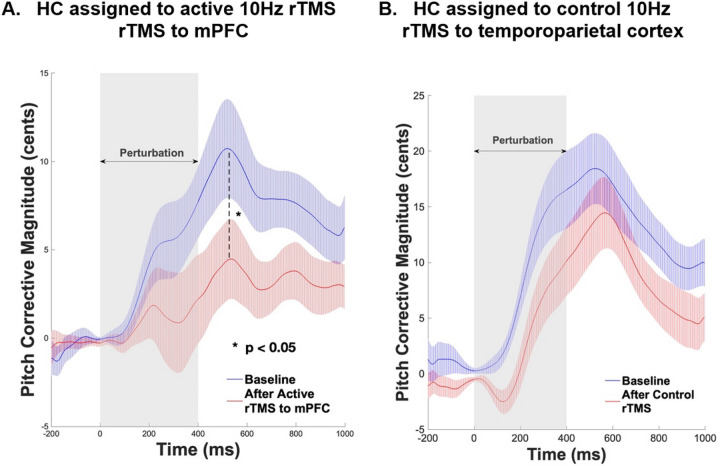
Mean pitch magnitude corrective responses during 100 cents pitch shifts, averaged across all participants at each time-point (i.e., baseline and after rTMS) in each condition shown for: **A.** participants assigned to active rTMS applied to mPFC and **B**. participants assigned to control rTMS. The grey rectangle illustrates the 400ms duration of the experiment-induced pitched perturbation. Participants began responding to the pitch perturbation shift at an average of 117ms after perturbation onset and peak response was reached at an average of 536ms after perturbation. Dashed lines represent the standard error of the corrective responses across all trials and participants for each condition and time-point.

Participants generated corrective behavioral responses to the 100 cents pitch perturbations in auditory feedback, most of which opposed the direction of the applied shift. On average, participants started responding to the applied perturbation at 117ms after perturbation onset and peak response was reached at 536ms after perturbation (Figure 3). Consistent with our hypothesis, compared to baseline and the control rTMS condition, enhancing mPFC excitability by rTMS induced participants to make significantly smaller corrective peak responses to the 100 cents pitch shift (F=5.77, p=0.04 and F=8.58, p =.01, respectively) (Figure 3). We did not find any difference in peak corrective responses in the control condition, after rTMS compared to baseline (F=1.2, p=.29). We also did not find any difference between the active rTMS and control rTMS conditions in the time to reach peak magnitude from perturbation onset either at baseline or after rTMS (all p’s >.05) (Figures 3A–B).

**Figure 4:**
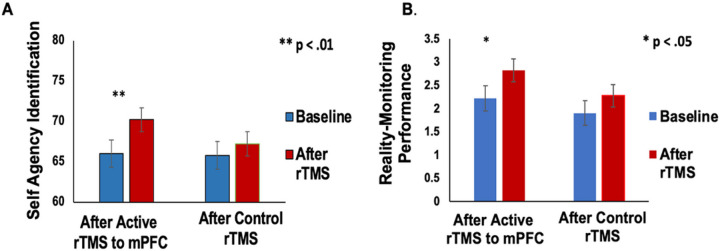
**A.** Repeated-measures ANOVA revealed HC had significant improvement in self agency judgments (i.e., % accuracy for self-generated information) that was observed only after HC completed active rTMS to mPFC compared to baseline, but not for HC in the control rTMS condition. **B**. Repeated-measures ANOVA revealed HC had significant improvement in overall reality monitoring performance (indexed by the d-prime score) that was also observed only after HC completed active rTMS to mPFC compared to baseline, but not for HC in the control rTMS condition.

Consistent with our hypothesis, on the reality monitoring task, enhancing mPFC excitability by rTMS induced significant improvement in self-agency judgments (F=6.0, p=.03), which contributed to improved reality monitoring performance (F=5.64, p=.04) (Figures 4A–B). We did not find any difference in the control condition, after rTMS compared to baseline, in either self-agency judgments (F=1.1, p=.31) or reality monitoring performance (F=3.6, p=.08).

**Figure 5:**
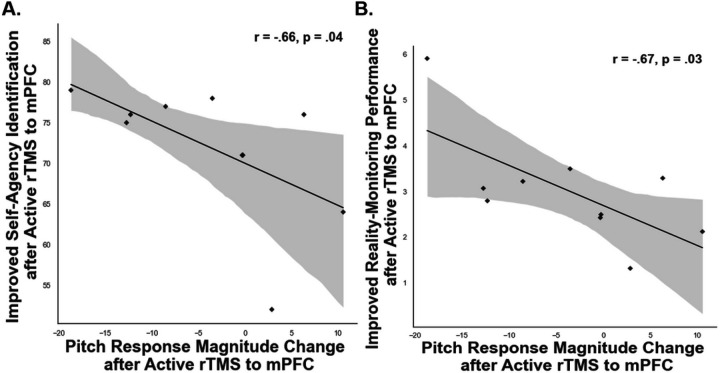
**A.** The scatterplot illustrates the significant negative correlation between the smaller change in peak corrective response to 100 cents pitch perturbations induced by 10Hz rTMS to mPFC with improved self-agency judgments (i.e., % accuracy for self-generated information) that was observed only after HC completed active rTMS to mPFC, but not for HC in the control rTMS condition. **B**. The scatterplot illustrates the significant negative correlation between the smaller change in peak corrective response to 100 cents pitch perturbations induced by 10Hz rTMS to mPFC with improved reality monitoring performance (indexed by the d-prime score) that was observed only after HC completed active rTMS to mPFC, but not for HC in the control rTMS condition.

Furthermore, we found a significant negative correlation between peak corrective responses to the 100 cents pitch perturbations with self-agency judgments (r=−.66, p=.04) and with reality-monitoring performance (r=−.67, p=.03) (Figures 5A–B). In other words, participants who made reduced corrective responses to compensate for their errors during pitch-induced perturbations demonstrated a greater sense of self-agency, which also potentiated their overall reality monitoring performance. Overall, these findings replicate and extend our prior findings (Subramaniam, 2021; Subramaniam et al., 2020; Subramaniam et al., 2018) and provide a causal basis in delineating the mechanism for how enhancing mPFC excitability by rTMS increased participants’ reliance on their internal self-predictions to guide their speech output, rather than reliance on external altered feedback to influence their speech output, which predicted and potentiated improved self-agency judgments on the reality-monitoring task.

We did not find any associations between pitch perturbation corrective responses and accuracy for externally-derived information on the reality monitoring task (p>.05). These results replicate and extend our prior results (Subramaniam, 2021; Subramaniam et al., 2018), in which we expected to only find correlations between self-agency judgments during reality monitoring with the peak magnitude of corrective responses during minimal pitch-induced perturbations experienced with the 100 cents pitch shift, as only these minimal perturbations are thought to maintain the sense of self-agency. Additionally, the time to reach peak corrective response was not associated with agency judgments during reality monitoring, revealing that only the magnitude of corrective responses provided reliable markers of self-agency judgments during the 100 cents pitch perturbation shifts (all p’s>.05). Finally, after rTMS to mPFC compared to baseline, Cohen’s d analyses yielded large effect sizes showing: (1) subjects made smaller pitch corrective responses, (2) subjects manifested improved self-agency judgements and (3) subjects manifested improved reality-monitoring performance (Cohen’s d = 0.73, .74, .72, respectively), as well as showing large effect sizes for the (4) correlation coefficients between rTMS-induced improved self-agency judgments and reality-monitoring performance with peak corrective responses during the 100 cents shift (Cohen’s d = 0.44 and 0.45, respectively). Together, these convergent findings across different types of analyses provide causal evidence for our hypothesis that the mPFC mediates the mechanisms underlying a unitary sense of self-agency that results from the ability to make *reliable* internal self-predictions about the outcomes of self-generated actions.

## Discussion

In this double-blinded RCT we found that, compared to control rTMS, enhancing mPFC excitability by 10Hz rTMS induced HC to: (1) make significantly smaller corrective responses to the 100 cents pitch shifts, (2) improve self-agency judgments and overall reality-monitoring performance, (3) manifest a significant association between smaller corrective responses on the speech monitoring task that predicted improved self-agency judgments during the reality monitoring task. The present findings were only observed for HC who completed active 10Hz rTMS targeting the mPFC, and were not observed in the control rTMS condition. Together, these findings indicate that the improvements in self-agency across both speech monitoring and reality monitoring tasks were specific to rTMS modulation of mPFC excitability, and cannot be attributed to practice task effects or general effects of rTMS.

Our findings are consistent with our prior studies in which we have previously shown a significant association in HC who made smaller pitch corrective responses on the speech monitoring task who also manifested a greater sense of self-agency on the reality monitoring task (Subramaniam, 2021; Subramaniam et al., 2018). Here, we now extend these prior correlative studies to delineate the causal mechanisms for how enhancing mPFC excitability by rTMS induced HC to make smaller corrective responses, reflecting their increased reliance on self-predictions to guide their speech output, which predicted and potentiated improved self-agency judgments on the reality-monitoring task.

Our results are also consistent with prior research indicating that subjects would produce corrective responses to oppose the error in their speech output that arises from the difference between the perceived and predicted auditory feedback (i.e., the prediction error), especially during minimal 100 cents pitch perturbations, as only these minimal pitch perturbations are thought to maintain the sense of self-agency (Behroozmand & Larson, 2011; Hain et al., 2001; Korzyukov et al., 2017). For example, participants are able to consciously judge larger pitch perturbations greater than 250 cents as non self-generated outcomes that are due to external changes in the environment, rather than being indicative of errors in their own speech output (i.e., self-agency) (Behroozmand & Larson, 2011; Hain et al., 2001; Korzyukov et al., 2017; Liu et al., 2011). The sensory outcome of self-generated actions is well-predicted, compared to external actions (Chang et al., 2013; Ford and Mathalon, 2012). Thus, typically the smaller the prediction error between the actual and predicted auditory feedback, the more likely the outcome will be attributed as a self-generated. However, we also believe that the sense of self-agency is not a direct result of this minimal prediction error. Rather, we believe that the mPFC produces an estimation of the ‘amount of reliance’ or the gain that needs to be placed on self-predictions and the prediction error in order to generate higher-order agency judgments. Here, we now extend this prior research on self-agency to another level (Korzyukov et al., 2017; Subramaniam, 2021; Subramaniam et al., 2018) by demonstrating that the mPFC represents one neural site that is able to causally modulate the gain of this prediction error (i.e., increase subjects’ confidence in their internal self-predictions about their speech outcome), to induce subjects to make smaller corrective responses. These smaller rTMS-induced corrective responses predicted improved conscious judgments of self-agency during reality monitoring. Together, the present findings delineate the causal impact of how modulating mPFC activity impacts self-agency, driven by improved reliance on self-prediction mechanisms.

The specific mPFC site we target with high-frequency rTMS is based on our convergent functional imaging studies (across fMRI and MEG imaging), in which HC showed increased activity within a specific anatomically and functionally consistent mPFC region that mediated self-agency (Subramaniam et al., 2019; Subramaniam et al., 2020; Subramaniam et al., 2012). Furthermore, we also found treatment-induced restoration of increased activity within this same mPFC site, which improved self-agency judgments, even in chronically-ill patients with psychiatric disorders (Subramaniam, 2021; Subramaniam et al., 2012; Subramaniam et al., 2017). Given these prior convergent data that mPFC supports mechanisms underlying self-agency (Subramaniam, 2021; Subramaniam et al., 2019; Subramaniam et al., 2020; Subramaniam et al., 2012; Subramaniam et al., 2017), here, we implemented rTMS to examine the causal mechanisms of whether modulating mPFC excitability not only modulates self-agency, but also improves self-prediction mechanisms that lead to the experience of self-agency on two distinct reality and speech monitoring tasks. In our previous rTMS study (Subramaniam et al., 2020), after pilot testing different TMS dosages targeting mPFC, we also demonstrated the optimal dosage (i.e., the best combination of rTMS parameters) that yielded maximum comfort for subjects while inducing significant neuromodulatory effects in the targeted mPFC site that we use here in the present application. For example, we found that rTMS protocols targeting mPFC for longer stimulation durations and higher frequencies (e.g. 20Hz) reduced tolerability ratings but did not further enhance self-agency judgments, compared to 10Hz rTMS. Cognitive efficacy and safety of targeting the prefrontal cortex with these TMS dosage parameters have also been validated in other studies without a single participant showing adverse events (Bentwich et al., 2011; Lee et al., 2016; Rabey et al., 2013). In the present study, while TMS did induce some discomfort caused by muscle twitches and tingling during the session, we did not observe any side-effects beyond the rTMS session in any subject or any adverse event (e.g. pain, headaches, seizures) with the present rTMS dosage parameters. All participants who began rTMS, completed the session and all post-rTMS assessments. Overall, this is the first causal proof-of-concept study to indicate that high-frequency 10Hz rTMS targeting mPFC was not only well-tolerated by participants, but also induced significant improvements in self-agency, that was driven by HC making improved self-predictions during a speech monitoring task, which potentiated improved self-agency judgments on a reality monitoring task.

We also clarify here that we are not stating that the mPFC represents the only neural correlate of self-agency. For example, prior research has shown that increased neural activity in other regions (e.g. motor regions such as the paracentral lobule/supplementary motor area and basal ganglia), have been observed only prior to self-initiated actions (but not externally-perceived actions), leading to the experience of self-agency (Cunnington et al., 2002; Khalighinejad et al., 2018). On the other hand, regions such as the posterior temporoparietal cortex which is located at a distant site from the active mPFC rTMS site, and thought to underlie general attention/salience to sensory information but considered to be an independent site outside of the direct self-agency network, represented an ideal control rTMS site (Behrmann et al., 2004; Constantinidis and Steinmetz, 2005; Shomstein and Yantis, 2006). Indeed, as confirmed by our hypotheses, we did not find any changes in either corrective responses during speech monitoring, self-agency judgments during reality monitoring or a significant association between corrective responses with self-agency judgments after HC completed the control rTMS. For both control and active rTMS conditions, HC are given the same rationale for participating in the study, engage in the same tasks from pre-to-post rTMS, and complete rTMS conducted with the same rTMS parameters, with only the rTMS target site being different. Thus, overall, these findings indicate that the mPFC is representative of one neural site whose excitation can causally modulate self-agency, and that these effects cannot be attributable to general rTMS effects or practice effects on the task.

In summary, we provide a novel perspective for investigating causal mechanisms underlying self-agency within two distinct speech monitoring and reality monitoring frameworks, based on one’s own self-predictions about the expected outcome of one’s own actions. In this first-of-its-kind multimodal study, we now deliver robust convergent evidence across two different reality and speech monitoring tasks, revealing that the mPFC represents one critical region that improves self-prediction mechanisms that lead to a unitary experience of self-agency. The present findings not only provide innovative functional biomarkers for understanding the underlying the neural basis of self-agency, but also provide the first step toward applying precision-medicine guided neuromodulation targets within this specific mPFC site that mediates self-agency. Such precision-medicine approaches in which we use convergent multimodal functional imaging data (across fMRI and MEG studies) as the basis for guiding neuromodulation target sites (Subramaniam et al., 2019; Subramaniam et al., 2020; Subramaniam et al., 2012), will enable the development of new TMS treatment interventions not only in HC but also in patients with psychosis-spectrum disorders who show cardinal impairments in self-agency that contribute to severe psychotic symptoms of hallucinations and delusions (Subramaniam, 2021; Subramaniam et al., 2012). In conclusion, our findings contribute to a larger body of literature on self-agency, and by specifying here that the mPFC represents a causal neural site that modulates self-agency, the present research creates a path towards developing new neuromodulation treatments interventions to improve self-agency that will be particularly useful for patients with psychosis disorders who exhibit severe impairments in self-agency.

## Figures and Tables

**Table 1.** Demographics (mean, SD) of Healthy Control Participants (HC)

	HC in Active rTMS to mPFC	HC in Control rTMS	p value
**Age (years)**	41 (16)	44 (18)	.60
**Gender**	10M, 5F	10M, 5F	1.0
**Education (years)**	17 (2.2)	17 (1.8)	.86

## Data Availability

Behavioral data and code analyses will be made available from the corresponding author upon request.
